# Supplementary material for: Brief Warm and Aldo-Keto Reductase Family AspiAKR1B1 Contribute to Cold Adaptation of Aleurocanthus spiniferus
Source: Insects. 2025 Jan 2;16(1):38. doi: 10.3390/insects16010038 (PMC11765982; doi:10.3390/insects16010038)
Supplement: Supplementary file 1 [file insects-16-00038-s001.zip › insects-3353887-supplementary.pdf]

Table S1. Primers used in this study.

Primer names	Primer sequence (5'–3')	Remarks
AspiAKR1A1-F	AAGCTTCTTCGTGGCGGTAA	qPCR
AspiAKR1A1-R	CTTCGTCGATGACGTCAGGT	
AspiAKR1A2-F	ACAGACCCGAAAGCGTTGAA	
AspiAKR1A2-R	CCGCACCCAGATTTTCTCCT	
AspiAKR1A3-F	TGGAGATGCAGTGGACAAGC	
AspiAKR1A3-R	AGCGTTCAGGATGGTTTCGT	
AspiAKR1B1-F	GCCAAGCTGTGAAAGATGCC	
AspiAKR1B1-R	TCTTCCCTTTTGACGGCTCC	
AspiAKR1B2-F	TGGATTGGGAACCTGGAAGC	
AspiAKR1B2-F	AGCAATAGCCTCGCCAACTT	
AspiAKR1B3-F	TGAGAAGGAAATCGGCGAGG	
AspiAKR1B3-R	GCTTCAAGCCCACTTCAACG	
qRPS28-F	AACCAGGACAAGTAGGCCAAG	
qRPS28-R	GCCTCGCCGATTTCCTTCTC	
AspiAKR1B1-F	TGCCATCCTTACTTGAGCCA	RNAi
AspiAKR1B1-R	CCATTCCAAGGCACAGATGC	
AspiAKR1B1-T7F	TAATACGACTCACTATAGGTGCCATCCTTACTTGAGCCA	
AspiAKR1B1-T7R	TAATACGACTCACTATAGGCCATTCCAAGGCACAGATGC	
dslta-T7F	TAATACGACTCACTATAGGCACCCTCTCCACGAATTG	
dslta-T7R	TAATACGACTCACTATAGGTAGAAGATGCTGCTGTTTCA	
